# Vitamin D metabolism pathway polymorphisms are associated with efficacy and safety in patients under anti-PD-1 inhibitor therapy

**DOI:** 10.3389/fimmu.2022.937476

**Published:** 2022-09-12

**Authors:** Jianquan Luo, Huiqing Chen, Fang Ma, Chenlin Xiao, Bao Sun, Yiping Liu, Haoneng Tang, Yue Yang, Wenhui Liu, Zhiying Luo

**Affiliations:** ^1^ Department of Pharmacy, The Second Xiangya Hospital, Central South University, Changsha, China; ^2^ Institute of Clinical Pharmacy, Central South University, Changsha, China; ^3^ Department of Oncology, The Second Xiangya Hospital, Central South University, Changsha, China; ^4^ Department of Laboratory Medicine, The Second Xiangya Hospital, Central South University, Changsha, China; ^5^ Department of Spine Surgery, The Second Xiangya Hospital of Central South University, Changsha, China

**Keywords:** immune checkpoint inhibitors, anti-PD-1 inhibitors, immune-related adverse effects, polymorphism, vitamin D

## Abstract

**Aim:**

Vitamin D (VitD) signaling has been increasingly investigated for its role in stimulating the innate and adaptive immune systems and suppressing inflammatory responses. Therefore, we examined the associations between VitD-related genetic polymorphisms, plasma 25-hydroxyvitamin D (25(OH)D), and the efficacy and safety of immune checkpoint inhibitors (ICIs).

**Patients and methods:**

A total of 13 single-nucleotide polymorphisms (SNPs) in VitD metabolic pathway genes were genotyped in 343 cancer patients receiving ICI treatment using the MassARRAY platform. In 65 patients, the associations between plasma 25(OH)D levels and ICI treatment outcomes were investigated further.

**Results:**

We found that the *CYP24A1* rs6068816TT and rs2296241AA genotypes were significantly higher in patients who responded to ICIs. Furthermore, patients with higher plasma 25(OH)D levels had a better treatment response. The distribution of allele and genotype frequencies showed that three SNPs (rs10877012, rs2762934, and rs8018720) differed significantly between patients who had immune-related adverse events (irAEs) and those who did not. There was no statistically significant relationship between plasma 25(OH)D levels and the risk of irAEs.

**Conclusion:**

In summary, our findings showed that genetic variations in the VitD metabolism pathway were associated with ICI treatment outcomes, and VitD supplementation may be useful in improving ICI treatment efficacy.

## Introduction

Immune checkpoint inhibitors (ICIs) are a novel class of drugs that target the programmed death ligand-1 (PD-L1)/programmed cell death protein-1 (PD-1) pathway and have been approved as first-line therapy for serious cancers (1). ICIs are essentially humanized monoclonal antibodies that can activate T cells and relieve the immune system from recognizing and assaulting cancer cells. Successful immunotherapy-induced anti-tumor immune responses require CD8^+^ and CD4^+^ T cells (2, 3). Because of the unsatisfactory efficacy of ICI monotherapy, ICIs combined with chemotherapy, radiotherapy, or anti-angiogenesis therapy have been approved as successful first-line therapy for several malignant tumors regardless of the PD-L1 expression level in tumor tissues (4). Investigating the mechanisms of insensitivity to immunotherapy has emerged as one of the most important challenges in cancer immunotherapy.

Paradoxically, combination therapy is often associated with a high incidence of immune-related adverse events (irAEs) (5, 6). The unleashed immune response could promote T-cell activation and autoimmunity, resulting in many systemic autoinflammatory reactions (7). IrAEs manifest differently in different patients, with some developing irAEs in a single organ and others developing irAEs in multiple organs (8). Several of these irAEs are self-limiting and easily manageable. Others may limit treatment, causing interruption that will require treatment with methylprednisolone or tumor necrosis factor-α antibody or even directly threaten life (9). Recent studies indicate that irAEs likely result from abnormal T- and B-cell activation and an overall increased inflammatory response, resembling the hyperimmune responses observed in autoimmune patients (10). Undoubtedly, the mechanisms of irAEs are complex and not fully understood. Because irAEs limit the therapeutic benefits of ICIs, identifying and investigating potential biomarkers that can predict the efficacy and safety of ICIs have received much attention in recent years.

Vitamin D (VitD) signaling has been increasingly investigated for its non-classical actions in stimulating the innate and adaptive immune systems and suppressing inflammatory responses (11, 12). It is known that VitD deficiency decreases the numbers of CD4+ and CD8+ T lymphocytes, while VitD supplementation increases CD4+ lymphocytes. In the direct and indirect pathways, VitD can induce and stimulate T-regulatory cells (Tregs), which can suppress proinflammatory responses by other immune cells and prevent exaggerated or autoimmune reactions (13). Similarly, VitD suppresses the tumor microenvironment by increasing the Treg/T-helper 17 (Th-17) cell ratio (14, 15). VitD has been shown to induce the expression of PD-L1 on human gut epithelial cells and PD-1 on immune cells in patients with inflammatory bowel diseases (16, 17). Numerous studies in the last decade have linked VitD deficiency and genetic polymorphisms in genes involved in the VitD metabolism pathway to an increased risk of several autoimmune diseases and cancers (18–20).

Recent studies suggest that the genetic background of patients receiving ICIs could play a role in susceptibility to irAEs (21). Several single-nucleotide polymorphisms (SNPs) located in genes related to VitD metabolism have been linked to plasma 25(OH)D levels and immune disease (22). Given the immunoregulatory activity of VitD, we hypothesize a possible link between polymorphisms associated with VitD physiological disposition and ICI treatment outcomes. We aimed to analyze the relationship between genetic variants underlying VitD metabolism (*VDR*, *CYP24A1*, *CYP27B1*, *CYP2R1*, *GC*, *DHCR7*, *RXRA*, and *SEC23A*) and the efficacy and safety in patients treated with anti-PD-1 inhibitors.

## Materials and methods

### Study population

A unicentric and retrospective study was conducted to elucidate the effect of genetic polymorphisms on the efficacy and safety of inter-individual differences. We collected the blood samples from patients receiving anti-PD-1 inhibitor (nivolumab or pembrolizumab) therapies regardless of treatment lines between October 2018 and January 2022 in the Department of Oncology, Second Xiangya Hospital, Central South University (Changsha, China). The Ethics Committee of Second Xiangya Hospital at Central South University (Changsha, China) approved this study, and all procedures followed the Declaration of Helsinki. The study was registered with the Chinese Clinical Trial Registry (ChiCTR2100045873). All patients provided written informed consent for blood banking and clinical information follow-up.

The inclusion criteria for patients are as follows: 1) age >18 years; 2) clinical symptoms, physical signs, imaging examination, and histologically or cytologically consistent with the diagnostic criteria for tumors; 3) treatment with anti-PD-1 inhibitor monotherapy or combination therapy at the recommended dose; and 4) no prior history of inflammatory or serious autoimmune diseases in the same affected organs.

### Collection of clinical variables and follow-up method

Electronic medical records were reviewed for demographics (such as age and sex), smoking and drinking status, primary tumor sites, histological types, Eastern Cooperative Oncology Group (ECOG) performance status (PS), PD-1 expression level, treatment strategies, and past medical history. All elements were abstracted and entered into a clinical data sheet. Patients in this study were prescribed five types of anti-PD-1 inhibitors (nivolumab, pembrolizumab, terelizumab, sindilizumab, and carrelizumab) and administered intravenously at a dose of 200 mg every 3 weeks as recommended. Other drugs, particularly broad-spectrum antibiotics, were also used. According to our previously published study (23), the follow-up lasted 6 months with regular clinic visits by an oncologist (Dr. Fang Ma) and two pharmacists (Pharm. Wenhui Liu and Pharm. Jianquan Luo).

The primary objective of this study was to determine the efficacy of an anti-PD-L1-based treatment strategy. According to Response Evaluation Criteria in Solid Tumors (RECIST) 1.1 criteria, the objective remission rate (ORR) and disease control rate (DCR) were used to assess the treatment efficacy. ORR was defined as the percentage of patients who achieved a complete response (CR) or partial response (PR) to treatment. The proportion of patients with CR, PR, or stable disease (SD) was defined as DCR. The second objective was irAEs, which were assessed and graded using the Common Terminology Criteria for Adverse Events version 4.0 (CTCAE4.0).

### Single-nucleotide polymorphism selection

The inclusion criterion for the candidate polymorphisms in our research is SNPs that may result in a functional alteration of the vitamin D metabolism pathway. The following were the selection criteria for the candidate SNPs: minor allele frequency (MAF) >1% in the Chinese population, with potential functions or associated with VitD concentration. Finally, we selected 13 SNPs from the VitD metabolism pathway, including *VDR* (rs1544410, rs731236, rs7975232, and rs2228570), *CYP24A1* (rs2296241, rs6068816, and rs2762934), *CYP27B1* (rs10877012), *CYP2R1* (rs2060793), *GC* (rs7041), *DHCR7* (rs12785878), *RXRA* (rs9409929), and *SEC23A* (rs8018720), which have been related to VitD circulating concentrations and immune disease. HaploReg showed that these polymorphisms were regulated by Enhancer histone marks, DNAse, proteins bound, motifs changed, and so on. However, the effects of the selected polymorphisms on gene expression remain unclear in previous studies.

### DNA extraction and genotyping

The Wizard Genomic DNA Purification kits (Promega, Madison, WI, USA) were used to extract genomic DNA from 2 ml of peripheral blood samples, according to the manufacturer’s protocol. Genotyping was performed using the SNP Sequenom MassARRAY platform (Bioyong Technologies Inc., Beijing, China). AssayDesigner (Ver. 3.1) designed the primers. The primer and probe sequences are shown in **Supplementary Table 1**. Subsequently, SNPs were genotyped using iPLEX Gold technology (Sequenom, San Diego, CA, USA), and automated data analysis was performed. SNPs with >1% MAF and >95% call rate have been sorted out for analysis. Additionally, 10% of randomly selected samples were retested using Sanger Sequencing, yielding a >99.9% concordance. The positive rates for these SNPs exceeded 90%, with some data missing due to competition in the genotyping reaction system in such a high-throughput technology.

### Measurement of plasma 25(OH)D

The plasma 25(OH)D level was measured at the Second Xiangya Hospital’s Department of Laboratory Medicine (Changsha, China). In this study, an additional 2 ml of peripheral blood was collected from 65 patients under ICI therapy using an EDTA anticoagulant tube. The plasma 25(OH)D level was determined using a chemiluminescent immunoassay (CLIA) by Roche, Elecsys 2010 (Basel, Switzerland). CLIA is the most commonly used clinical test for the VitD state. The Elecsys VitD total assay uses a VitD binding protein as a capture protein to bind vitamin D3 (25-OH) and vitamin D2 (25-OH). The measuring range was 3.00–70.0 ng/ml or 7.50–175 nmol/L (defined by the limit of detection and the maximum of the master curve). VitD deficiency is defined as a VitD (25-OH) concentration of ≤20.0 ng/ml (≤50.0 nmol/L). VitD insufficiency is recognized as 21.0–29.0 ng/ml.

### Statistical analyses

Both SPSS 19.0 (IBM Corp., Chicago, IL, USA) and Plink v1.07 (http://pngu.mgh.harvard.edu/purcell/plink/) were used to carry out the statistical analysis. All tests were two-tailed, and p-values <0.05 were considered a significant difference. Quantitative data were presented as mean values and standard deviation (SD). The chi-squared test or Fisher’s exact test was used to compare categorical data and evaluate the Hardy–Weinberg equilibrium (HWE). Direct counting was used to compare genotype and allele frequencies, and a t-test was used for between-group comparisons. The associations between polymorphisms and the risk of treatment outcomes were expressed as the odds ratios (ORs) and 95% confidence intervals (CIs).

## Results

### Clinical parameters and their impact on efficacy and safety of immune checkpoint inhibitors

This study included 343 qualified patients who received anti-PD-1 inhibitors regardless of treatment line and had an available blood sample. **Table 1** shows the demographic and baseline characteristics of the patients involved. The overall median age of the patients was 58.41 ± 10.63 years, with 283 (82.51%) being male. More than half of the patients (51.89%) had a history of smoking, and 30.03% had a history of drinking alcohol. Most of the enrolled patients had advanced malignant tumors (stage III–IV), particularly non-small cell lung cancer (NSCLC). Most patients had good health conditions (ECOG PS score ≤1). Most patients had PD-L1 expression levels tested before being administered with anti-PD-1 inhibitors, and approximately two-thirds (≥1%) of patients had positive PD-L1 expression levels. In 301 (87.75%) patients, anti-PD-1 inhibitors combined with chemotherapy or radiotherapy were the most frequently used first-line treatment strategy.

**Table 1:** Demographic and baseline characteristics of enrolled samples.

Characteristics	Patient count (N = 343)
**Age, years**	
Mean ± SD	58.41 ± 10.63
Sex	
Male Female	283 (82.51%) 60 (17.49%)
BMI, mean ± SD	22.20 ± 3.70
BMI < 18.5	36 (10.50%)
18.5 ≤ BMI ≤ 24.9	244 (71.14%)
25 ≤ BMI ≤ 29.9	59 (17.20%)
BMI > 30	4 (1.17%)
Smoke habit	178 (51.89%)
Drink habit	103 (30.03%)
Disease stage	
I–II	41 (11.95%)
III–IV	302 (88.05%)
Cancer type	
Non-small cell lung cancer	213 (62.10%)
Esophagus cancer	31 (9.04%)
nasopharyngeal carcinoma	14 (4.08%)
Malignant melanoma	5 (1.45%)
Other types	80 (23.32%)
ECOG PS score before treatment	
0	6 (1.70%)
1	320 (93.30%)
≥2	17 (5.0%)
Patients with PD-L1 expression level	245 (71.43%)
<1%	84 (34.28%)
1%–49%	79 (32.24%)
≥50%	82 (33.48%)
Anti-PD-1 plus chemotherapy or radiotherapy	301 (87.75%)
Anti-PD-1 monotherapy	42 (12.25%)
Treatment line	
First line therapy	242 (70.55%)
Second- or third-line therapy	101 (29.45%)

BMI, body mass index; ECOG PS score, Eastern Cooperative Oncology Group performance status.

**Figure 1:**
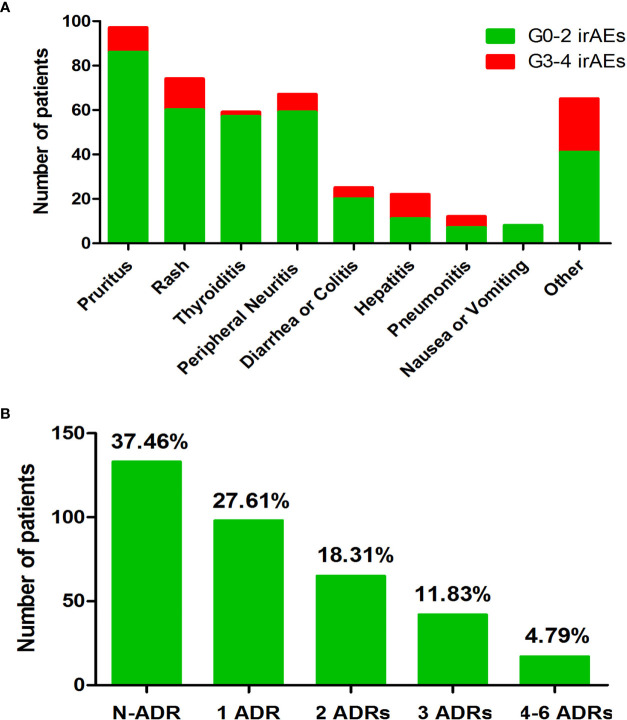
Overview of the irAEs that occurred during follow-up. **(A)** Number of patients of each type of irAE. **(B)** Number of irAEs that occurred during follow-up time. Note. N-ADR, number of patients without adverse drug response (ADR); 1 ADR, number of patients with one kind of ADR; 2 ADR, number of patients with two kinds of ADR; 3 ADR, number of patients with three kinds of ADR; 4–6 ADR, number of patients with four to six kinds of ADR; irAEs, immune-related adverse events.

The median follow-up duration for this cohort was 11.7 months [interquartile range, 7.2–16.5 months]. We considered therapy effective when patients were classified as CR, PR, or SD. Patients were evaluated as PD when their treatment was infective. Only 296 (86.30%) patients had available data to assess efficacy. Sixteen (4.66%) patients achieved CR, 129 (37.61%) patients achieved PR, 96 (27.99%) patients experienced SD, and 55 (16.03%) patients suffered PD. ORR was 70.26%, and DCR was 42.27%. **Table 2** illustrates the treatment efficacy of anti-PD-1 inhibitors in this study. During the follow-up period, a total of 215 patients developed irAEs, and most patients (159, 73.95%) presented with mild irAEs. The most frequently reported irAEs in our patient population were pruritus and/or rash, thyroid dysfunction, and peripheral neuritis, as shown in **Figure 1A**. **Figure 1B** shows that 120 patients had at least two types of irAEs. Most patients (159, 46.35%) experienced mild irAEs, and only 56 (16.33%) suffered from severe irAEs (grade 3–5).

**Table 2:** The evaluated treatment efficacy by RECIST1.1.

Primary endpoints (efficacy)	
Complete response	16 (4.66%)
Partial response	129 (37.61%)
Stable disease	96 (27.99%)
Progressive disease	55 (16.03%)
Not evaluable	47 (13.70%)
DCR	241 (70.26%)
ORR	145 (42.27%)
Second endpoints (safety)	343 (100%)
irAEs	215 (62.68%)
Severe irAEs (grades 3–5)	56 (16.33%)
Mild irAEs (grades 1–2)	159 (46.35%)
No immune-related adverse events	128 (37.32%)

RECIST, Response Evaluation Criteria in Solid Tumors; DCR, disease control rate; ORR, objective remission rate; irAEs, immune-related adverse events.

### Clinical parameters and their impact on efficiency

Five patients died due to disease progression, and we were unable to evaluate efficacy in 47 patients during follow-up. Because most patients were still alive at the end of the follow-up period, obtaining mean overall survival for patients was not feasible. In univariate analysis, positive factors in patients with good efficacy (DCR) included NSCLC diagnosis (p = 0.012), higher PD-L1 expression level (p = 0.006), and use of anti-PD-L1 inhibitors (p = 0.001) as first-line treatment. Negative factors, such as patients with a higher PS score (≥2) and ICI monotherapy, were associated with lower treatment efficacy. Our findings confirmed that the incidence of irAEs was associated with a more favorable prognosis (66.39% *vs*. 50%, p = 0.029). Age, sex, body mass index, smoking and drinking habits, or disease stage (p > 0.05) did not affect treatment efficacy. These findings are presented in **Supplementary Table 2**.

### Pharmacogenomic association of vitamin D pathway polymorphisms with the efficacy of immune checkpoint inhibitors

First, we genotyped 13 SNPs in 343 patients, and the complete list of candidate SNPs for our sample is shown in **Table 3**. The potential function of these polymorphisms was analyzed based on HaploReg. The allele frequencies in the studied sample were similar to the MAF value of CHS: Southern Han Chinese from the 1000 Genomes Project. The genotyping results revealed that each SNP had a higher than 95% call rate. One SNP (*VDR* rs2228570) (p < 0.05) was excluded from the subsequent analyses due to its deviation from HWE.

**Table 3:** Characteristics of studied SNPs from vitamin D metabolism pathway.

Gene	Chr	SNP	MAF (ref^a^)	Alleles	Location	Call rate (%)	p^b^
VDR	12	rs1544410	0.041 (0.087)	T:C	Intron variant	99.71	0.557
	12	rs731236	0.05 (0.078)	G:A	Synonymous variant	98.25	0.329
	12	rs7975232	0.31 (0.28)	A:C	Intron variant	97.38	0.98
	12	rs2228570	0.47 (0.42)	A:G	Missense variant	97.96	3E−04
CYP24A1	20	rs2296241	0.45 (0.41)	A:G	Synonymous variant	98.83	0.82
	20	rs6068816	0.37 (0.37)	T:C	Synonymous variant	96.79	0.18
	20	rs2762934	0.10 (0.092)	A:G	3′ prime UTR variant	98.25	0.34
CYP27B1	12	rs10877012	0.36 (0.36)	G:T	2KB Upstream Variant	97.08	0.54
CYP2R1	11	rs2060793	0.36 (0.34)	A:G	5′ prime UTR variant	98.54	0.04
GC	4	rs7041	0.29 (0.27)	C:A	Missense variant	99.13	0.26
DHCR7	11	rs12785878	0.42 (0.47)	T:G	5′ flanking	98.54	0.38
RXRA	9	rs9409929	0.20 (0.18)	A:G	NA	95.04	0.33
SEC23A	14	rs8018720	0.38 (0.40)	G:C	Missense variant	99.13	0.80

SNP, single-nucleotide polymorphism; MAF, minor allele frequency.

a ref indicates the MAF value of CHS from 1000 Genomes Project.

b p-Value for Hardy–Weinberg equilibrium analysis.

Second, we established a link between genotypes and treatment response (DCR). These findings are shown in **Supplementary Table 3**. Two SNPs (rs6068816 and rs2296241) were significantly associated with DCR (**Table 4**). The frequency of rs6068816T allele was significantly lower in the patients with ineffective responses than in those with effective responses (47.11% *vs*. 66.38%, OR (95% CI): 0.45 (0.29–0.69); p = 3.14E−4). The frequency of the rs2296241A allele in patients with ineffective treatment was significantly lower than in those with effective treatment (33.96% *vs*. 46.86%, OR (95% CI): 0.58 (0.37–0.91); p = 0.017). These two SNPs were also associated with DCR (p = 0.00058 and p = 0.048) after adjusting for baseline PS score, cancer type, treatment line, PD-L1 expression level, anti-PD-1 monotherapy, and treatment line. The linkage disequilibrium (LD) information presented here for the SNP pair (rs6068816 and rs2296241) is based on haplotype frequencies estimated using the expectation–maximization (EM) algorithm, R^2^ = 0.25, D′ = 0.73. This result showed that these two SNPs had a weak LD association.

**Table 4:** SNPs significantly associated with treatment efficacy.

SNP	Model	Genotype/allele	Ineffective (N = 54)	Effective (N = 215)	OR (95 CI)	p
rs6068816	Genotypic model	TT CT CC	10 (19.23%) 29 (55.77%) 13 (25.00%)	97 (41.28%) 118 (50.21%) 20 (8.51%)	Reference 0.42 (0.19–0.90) 0.16 (0.061–0.41)	5.06E−04 0.033 1.77E−4
	Dominant model	TT *vs*. CT+CC	10 (19.23%)/42 (80.77%)	97 (41.28%)/138 (58.72%)	0.34 (0.016–0.71)	0.003
	Recessive model	CC *vs*. TC+TT	13 (25.00%)/39 (75.00%)	20 (8.51%)/138 (91.49%)	0.28 (0.13–0.61)	0.003
	Allelic model	T *vs*. C	49 (47.12%)/55 (52.88%)	312 (66.38%)/158 (33.62%)	0.45 (0.29–0.69)	3.14E−4
rs2296241	Genotypic model	CC CA AA	21 (39.62%) 28 (52.83%) 4 (7.55%)	67 (28.03%) 120 (50.21%) 52 (21.76%)	Reference 1.34 (0.71–2.54) 4.07 (1.31–12.60)	0.029 0.41 0.012
	Dominant model	CC *vs*. CA+AA	21 (39.62%)/32 (60.38%)	67 (28.03%)/172 (71.97%)	1.68 (0.91–3.13)	0.10
	Recessive model	AA *vs*. CC+CA	4 (7.55%)/49 (92.45%)	52 (21.76%)/187 (78.24%)	0.29 (0.10–0.85)	0.019
	Allelic model	A *vs*. C	36 (33.96%)/70 (66.04%)	224 (46.86%)/254 (53.14%)	0.58 (0.37–0.91)	0.017

SNP, single-nucleotide polymorphism.

### Pharmacogenomic association of vitamin D pathway polymorphisms with the safety of immune checkpoint inhibitors

Except for the disease stage, the demographic and baseline characteristics were nearly identical between patients with or without irAEs (as shown in **Supplementary Table 4**). As shown in **Supplementary Table 5**, the association analysis results in this cohort revealed that two SNPs (rs10877012 and rs8018720) were significantly associated with the development of irAEs. The allele and genotype frequency distribution showed that three SNPs differed significantly between groups. In *CYP27B1* gene, TT genotype carriers had a significantly lower risk of irAEs than rs10877012 GG+GT genotype carriers (50.41% *vs*. 33.49%, OR (95% CI): 0.51 (0.32–0.80); p = 0.0037). The G allele frequency was significantly lower in patients who did not experience irAEs than in those who did (29.34% *vs*. 40.09%, OR (95% CI): 0.61 (0.44–0.86); p = 0.0057). In *CYP24A1* gene, the rs2762934G allele was significantly lower in patients with irAEs compared to patients without irAEs (92.58% *vs*. 87.80%, p = 0.043). For *SEC23A* rs8018720, GC genotype carriers were more likely to develop irAEs (53.05% *vs*. 39.37%, p = 0.049). These results are presented in **Table 5**.

**Table 5:** SNPs significantly associated with the development of irAEs.

SNP	Model	Genotype/allele	N-irAEs group (N = 128)	irAE group (N = 215)	OR (95 CI)	p
rs10877012	Genotypic model	GG GT TT	11 (9.09%) 49 (40.50%) 61 (50.41%)	30 (13.95%) 110 (51.16%) 72 (33.49%)	Reference 0.82 (0.38–1.77) 0.43 (0.20–0.93)	0.012 0.70 0.045
	Dominant model	GG+GT *vs*. TT	60 (49.59%)/61 (50.41%)	140 (66.51%)/72 (33.49%)	0.51 (0.32–0.80)	0.0037
	Recessive model	GG *vs*. GT+TT	11 (9.09%)/110 (90.91%)	30 (14.15%)/182 (85.85%)	0.61 (0.29–1.26)	0.22
	Allelic model	G *vs*. T	71 (29.34%)/171 (70.66%)	170 (40.09%)/254 (59.91%)	0.62 (0.44–0.86)	0.0055
rs2762934	Allelic model	A *vs*. G	19 (7.42%)/237 (92.58%)	51 (12.20%)/367 (87.80%)	0.58 (0.33–1.00)	0.043
rs8018720	Genotypic model	CC CG GG	54 (42.52%) 50 (39.37%) 23 (18.11%)	74 (34.74%) 113 (53.05%) 26 (12.21%)	Reference 1.65 (1.02–2.67) 0.82 (0.43–1.60)	0.041 0.049 0.61

### The relationship between plasma 25(OH)D concentration and immune checkpoint inhibitor clinical outcomes

Finally, plasma 25(OH)D levels were measured in 65 patients in this study, with a mean age of 57.56 ± 9.43 years. Eight patients responded to ICIs, and 41 patients developed irAEs. The plasma level of 25(OH)D was significantly higher in patients with effective responses (44.56 ± 13.15 ng/ml) than in patients with ineffective outcomes (33.67 ± 11.36 ng/ml) (p = 0.001). In contrast, no difference in 25(OH)D levels was found between patients with and without irAEs (43.81 ± 12.15 *vs*. 38.11 ± 12.43 ng/ml, p = 0.38).

## Discussion

Recently, VitD signaling has been increasingly investigated for its non-classical actions in stimulating innate immunity and suppressing inflammatory responses. In this retrospective study, we examined the association between polymorphisms in the VitD metabolism pathway and the efficacy and safety of anti-PD-L1 treatment. We identified two SNPs in *CYP24A1* gene linked to a higher likelihood of treatment response. Furthermore, three SNPs were statistically related to the risk of irAEs.

Previous studies have shown that clinical parameters, such as PD-L1 expression level, tumor histology, and anti-PD-1 monotherapy, are significantly associated with ICI treatment efficacy (24, 25). Our findings are consistent with those studies, implying that our findings are reliable. For instance, our study found that patients with NSCLC who had lower PS scores before ICI treatment, high PD-L1 expression levels, combination therapy, and ICIs as first-line therapy were more likely to benefit from ICI treatment. Furthermore, we confirmed that patients who respond to ICIs are more likely to develop irAEs. This finding raises the possibility of shared genetic relationships between treatment-related toxicity and efficacy. One previous study, for example, found that two human leukocyte antigen (HLA) alleles (HLA-DRB1*11:01 and HLA-DQB1*03:01) are predisposed to autoimmune diseases and are associated with an increased risk of developing pruritus or colitis during immunotherapy (26). Similarly, HLA genotyping was previously performed in patients with melanoma and NSCLC treated with ICIs; patients with HLA-B44 had longer survival, whereas those with HLA-B62 had worse disease outcomes (27).

VitD comprises a group of structurally related fat-soluble compounds that regulate over 200 genes and are essential for a wide range of physiological processes (28). VitD is first hydroxylated at the 25 position to 25(OH)D by *CYP27A1* and *CYP2R1*, 25(OH)D is considered active VitD, and its plasma concentrations can be used to determine the VitD status of patients. In the kidney, 1-hydroxylase (encoded by *CYP27B1*) hydroxylates to 1,25-dihydroxy VitD [1, 25(OH)2D]. Finally, 24-hydroxylase (*CYP24A1*) converts the active forms of 25(OH)D and 1,25(OH)2D into inactive forms (13). Previously, studies found that variants near genes involved in VitD transport could regulate VitD levels, and the presence of SNPs might influence autoimmune disease susceptibility by causing VitD deficiency (29, 30). Previous research has rarely reported on the role of genes involved in the VitD metabolic pathway in ICI treatment. According to one recently published randomized controlled trial, supplementing with VitD at a dose of 2,000 IU/day for approximately 5 years resulted in a lower incidence of autoimmune disease (22%) than placebo (31). Osama et al. first identified VitD as a protective factor against the development of ICI-induced colitis (32). Furthermore, 1,25(OH)2D has been shown to trigger tumor resistance by maintaining elevated PD-L1 and PD-L2 signaling in the tumor microenvironment, suppressing T cell-mediated anti-tumor immunity (33).

In this current study, we found that *CYP24A1* rs6068816 and rs2296241 polymorphisms were significantly associated with ICI efficacy. After adjustment for clinical factors, patients with the rs6068816T and rs2296241A alleles were more likely to benefit from immunotherapy. Moreover, we found that patients with higher plasma levels of 25(OH)D responded better to ICIs. One recent study showed that the rs6068816T allele is associated with higher levels of 25(OH)D concentration and a lower risk of NSCLC, whereas no such association was found for the rs2296241 allele (34). However, inconsistent results were found in other studies, which found that both the rs6068816 and rs2296241 polymorphisms were not associated with 25(OH)D concentration levels (35, 36). Because of a synonymous polymorphism, rs6068816 and rs2296241 cannot alter the amino acid sequence of *CYP24A1*. Therefore, we hypothesized that rs6068816 and rs2296241 might be involved in the treatment efficacy of ICIs by affecting VitD status, and the mechanism needed to be validated by more rigorous studies with a larger sample size and a different ethnic population.

Despite the lack of association between 25(OH)D level and the risk of irAEs, our study found that several mutations, including *CYP27B1* rs10877012, *CYP24A1* rs2762934, and *SEC23A* rs8018720, significantly reduced the risk of irAEs. *CYP27B1* rs10877012 is located in the promoter of *CYP27B1*, and the rs10877012GG genotype is associated with significantly higher serum 25(OH)D levels compared to GT/TT genotypes (37). Rs2762934 is found in the 3′ untranslated region of *CYP24A1* gene, and AA/AG genotypes are associated with an increased risk of VitD deficiency (38). A previous genome-wide association study identified that rs8018720 in *SEC23A* is significantly associated with serum 25(OH)D concentration (39). However, further studies failed to validate this association (40, 41). These inconsistencies might be explained by the genetic backgrounds of different disease types, sample sizes, and races.

In conclusion, our findings demonstrated that NSCLC patients with lower PS scores had high PD-L1 expression levels, combination therapy, and ICIs as first-line therapy were more likely to benefit from ICI treatment. The pharmacogenomics results revealed that *CYP24A1* rs6068816 and rs2296241 polymorphisms act as independent beneficial factors in the treatment response of ICIs. The allele and genotype frequency distribution showed three SNPs (rs10877012, rs2762934, and rs8018720) associated with the risk of irAEs. Furthermore, we found that plasma VitD levels were significantly higher in patients who responded to ICIs. The abo-e findings implied that investigating the role of VitD in the treatment outcomes of ICIs was of great clinical importance. However, our study also has limitations. Despite enrolling a large number of patients, the population studied for the role of plasma VitD concentration on ICI treatment was relatively small. Second, only DCR and ORR were used to assess ICI treatment efficacy. The role of genetic factors and plasma VitD concentration in other efficacy-related indicators (progression-free survival, overall survival, and so on) deserves further study. Finally, because the genotyping and chemiluminescent immunoassay tests were not performed on the same samples, we could not further investigate the relationship between gene polymorphism and 25(OH)D level. The precise role of VitD metabolic pathway genes and plasma VitD levels in ICI treatment outcomes needs to be explored further in future repetitive and functional studies.

## Data availability statement

The original contributions presented in the study are included in the article/**Supplementary Material**. Further inquiries can be directed to the corresponding authors.

## Ethics statement

This study was reviewed and approved by the Ethic Committee of Second Xiangya Hospital at Central South University. The patients/participants provided their written informed consent to participate in this study.

## Author contributions

All authors made a significant contribution to the work reported, whether in the conception, study design, execution, acquisition of data, analysis, and interpretation, or in all these areas; took part in drafting, revising, or critically reviewing the article; gave final approval of the version to be published; have agreed on the journal to which the article has been submitted; and agree to be accountable for all aspects of the work.

## Funding

This work was supported by the National Natural Scientific Foundation of China (82003883) and Natural Science Foundation of Hunan Province China (Grant Nos. 2020JJ5822, 2021JJ40847).

## Acknowledgments

The authors thank all the patients and their families. We thank the support from the doctors of the Department of Oncology, the Second Xiangya Hospital of Central South University.

## Conflict of interest

The authors declare that the research was conducted in the absence of any commercial or financial relationships that could be construed as a potential conflict of interest.

## Publisher’s note

All claims expressed in this article are solely those of the authors and do not necessarily represent those of their affiliated organizations, or those of the publisher, the editors and the reviewers. Any product that may be evaluated in this article, or claim that may be made by its manufacturer, is not guaranteed or endorsed by the publisher.

## References

[1] Korman AJ Garrett-Thomson SC Lonberg N . The foundations of immune checkpoint blockade and the ipilimumab approval decennial. Nat Rev Drug Discov (2021) 21:509–28. doi: 10.1038/s41573-021-00345-8 34937915

[2] Lee J Lozano-Ruiz B Yang FM Fan DD Shen L González-Navajas JM . The multifaceted role of Th1, Th9, and Th17 cells in immune checkpoint inhibition therapy. Front Immunol (2021) 12:625667. doi: 10.3389/fimmu.2021.625667 33777008 PMC7994325

[3] Liu R Yang F Yin JY Liu YZ Zhang W Zhou HH . Influence of tumor immune infiltration on immune checkpoint inhibitor therapeutic efficacy: A computational retrospective study. Front Immunol (2021) 12:685370. doi: 10.3389/fimmu.2021.685370 34220837 PMC8248490

[4] Langer CJ Gadgeel SM Borghaei H Papadimitrakopoulou VA Patnaik A Powell SF . Carboplatin and pemetrexed with or without pembrolizumab for advanced, non-squamous non-small-cell lung cancer: a randomised, phase 2 cohort of the open-label KEYNOTE-021 study. Lancet Oncol (2016) 17:1497–508. doi: 10.1016/S1470-2045(16)30498-3 PMC6886237 27745820

[5] Horn L Mansfield AS Szczęsna A Havel L Krzakowski M Hochmair MJ . First-line atezolizumab plus chemotherapy in extensive-stage small-cell lung cancer. N Engl J Med (2018) 379:2220–9. doi: 10.1056/NEJMoa1809064 30280641

[6] Gandhi L Rodríguez-Abreu D Gadgeel S Esteban E Felip E De Angelis F . Pembrolizumab plus chemotherapy in metastatic non-Small-Cell lung cancer. N Engl J Med (2018) 378:2078–92. doi: 10.1056/NEJMoa1801005 29658856

[7] Baxi S Yang A Gennarelli RL Khan N Wang Z Boyce L . Immune-related adverse events for anti-PD-1 and anti-PD-L1 drugs: systematic review and meta-analysis. BMJ (Clinical Res ed.) (2018) 360:k793. doi: 10.1136/bmj.k793 PMC5851471 29540345

[8] Martins F Sofiya L Sykiotis GP Lamine F Maillard M Fraga M . Adverse effects of immune-checkpoint inhibitors: epidemiology, management and surveillance. Nat Rev Clin Oncol (2019) 16:563–80. doi: 10.1038/s41571-019-0218-0 31092901

[9] Kawai T Taguchi S Nakagawa T Kamei J Nakamura Y Obinata D . Impact of immune-related adverse events on the therapeutic efficacy of pembrolizumab in urothelial carcinoma: a multicenter retrospective study using time-dependent analysis. J Immunother Cancer (2022) 10:e003965. doi: 10.1136/jitc-2021-003965 35210308 PMC8883255

[10] Geisler AN Phillips GS Barrios DM Wu J Leung DYM Moy AP . Immune checkpoint inhibitor-related dermatologic adverse events. J Am Acad Dermatol (2020) 83:1255–68. doi: 10.1016/j.jaad.2020.03.132 PMC7572894 32454097

[11] Kamen DL Tangpricha V Vitamin D . And molecular actions on the immune system: modulation of innate and autoimmunity. J Mol Med (Berlin Germany) (2010) 88:441–50. doi: 10.1007/s00109-010-0590-9 PMC2861286 20119827

[12] Arora J Wang J Weaver V Zhang Y Cantorna MT . Novel insight into the role of the vitamin d receptor in the development and function of the immune system. J Steroid Biochem Mol Biol (2022) 219:106084. doi: 10.1016/j.jsbmb.2022.106084 35202799 PMC8995385

[13] Prietl B Treiber G Pieber TR Amrein K Vitamin D . And immune function. Nutrients (2013) 5:2502–21. doi: 10.3390/nu5072502 PMC3738984 23857223

[14] Sassi F Tamone C D'Amelio P Vitamin D . Nutrient, hormone, and immunomodulator. Nutrients (2018) 10:1656. doi: 10.3390/nu10111656 PMC6266123 30400332

[15] Zeitelhofer M Adzemovic MZ Gomez-Cabrero D Bergman P Hochmeister S N'Diaye M . Functional genomics analysis of vitamin d effects on CD4+ T cells *in vivo* in experimental autoimmune encephalomyelitis. Proc Natl Acad Sci USA (2017) 114:E1678–e1687. doi: 10.1073/pnas.1615783114 28196884 PMC5338504

[16] Morita M Okuyama M Akutsu T Ohdaira H Suzuki Y Urashima M . Vitamin d supplementation regulates postoperative serum levels of PD-L1 in patients with digestive tract cancer and improves survivals in the highest quintile of PD-L1: A *Post hoc* analysis of the AMATERASU randomized controlled trial. Nutrients (2021) 13:1987. doi: 10.3390/nu13061987 34207794 PMC8228230

[17] Dimitrov V Bouttier M Boukhaled G Salehi-Tabar R Avramescu RG Memari B . Hormonal vitamin d up-regulates tissue-specific PD-L1 and PD-L2 surface glycoprotein expression in humans but not mice. J Biol Chem (2017) 292:20657–68. doi: 10.1074/jbc.M117.793885 PMC5733602 29061851

[18] Yang CY Leung PS Adamopoulos IE Gershwin ME . The implication of vitamin d and autoimmunity: a comprehensive review. Clin Rev Allergy Immunol (2013) 45:217–26. doi: 10.1007/s12016-013-8361-3 PMC6047889 23359064

[19] Stucci LS D'Oronzo S Tucci M Macerollo A Ribero S Spagnolo F . Vitamin d in melanoma: Controversies and potential role in combination with immune check-point inhibitors. Cancer Treat Rev (2018) 69:21–8. doi: 10.1016/j.ctrv.2018.05.016 29864718

[20] Guo Z Huang M Fan D Hong Y Zhao M Ding R . Association between vitamin d supplementation and cancer incidence and mortality: A trial sequential meta-analysis of randomized controlled trials. Crit Rev Food Sci Nutr (2022) 62:1–15. doi: 10.1080/10408398.2022.2056574 35352965

[21] Abdel-Wahab N Diab A Yu RK Futreal A Criswell LA Tayar JH . Genetic determinants of immune-related adverse events in patients with melanoma receiving immune checkpoint inhibitors. Cancer Immunol Immunother CII (2021) 70:1939–49. doi: 10.1007/s00262-020-02797-0 PMC10992432 33409738

[22] Bahrami A Sadeghnia HR Tabatabaeizadeh SA Bahrami-Taghanaki H Behboodi N Esmaeili H . Genetic and epigenetic factors influencing vitamin d status. J Cell Physiol (2018) 233:4033–43. doi: 10.1002/jcp.26216 29030989

[23] Liu W Ma F Sun B Liu Y Tang H Luo J . Intestinal microbiome associated with immune-related adverse events for patients treated with anti-PD-1 inhibitors, a real-world study. Front Immunol (2021) 12:756872. doi: 10.3389/fimmu.2021.756872 34975845 PMC8716485

[24] van Not OJ de Meza MM van den Eertwegh AJM Haanen JB Blank CU Aarts MJB . Response to immune checkpoint inhibitors in acral melanoma: A nationwide cohort study. Eur J Cancer (Oxford Engl 1990) (2022) 167:70–80. doi: 10.1016/j.ejca.2022.02.026 35395553

[25] Loo K Smithy JW Postow MA Betof Warner A . Factors determining long-term antitumor responses to immune checkpoint blockade therapy in melanoma. Front Immunol (2021) 12:810388. doi: 10.3389/fimmu.2021.810388 35087529 PMC8787112

[26] Hasan Ali O Berner F Bomze D Fässler M Diem S Cozzio A . Human leukocyte antigen variation is associated with adverse events of checkpoint inhibitors. Eur J Cancer (Oxford Engl 1990) (2019) 107:8–14. doi: 10.1016/j.ejca.2018.11.009 30529903

[27] Chowell D Morris LGT Grigg CM Weber JK Samstein RM Makarov V . Patient HLA class I genotype influences cancer response to checkpoint blockade immunotherapy. Sci (New York NY) (2018) 359:582–7. doi: 10.1126/science.aao4572 PMC6057471 29217585

[28] Carlberg C . Vitamin d and its target genes. Nutrients (2022) 14:1354. doi: 10.3390/nu14071354 35405966 PMC9003440

[29] Wang TJ Zhang F Richards JB Kestenbaum B van Meurs JB Berry D . Common genetic determinants of vitamin d insufficiency: a genome-wide association study. Lancet (London England) (2010) 376:180–8. doi: 10.1016/S0140-6736(10)60588-0 PMC3086761 20541252

[30] Zhang TP Li HM Huang Q Wang L Li XM . Vitamin d metabolic pathway genes polymorphisms and their methylation levels in association with rheumatoid arthritis. Front Immunol (2021) 12:731565. doi: 10.3389/fimmu.2021.731565 34925313 PMC8677352

[31] Hahn J Cook NR Alexander EK Friedman S Walter J Bubes V . And marine omega 3 fatty acid supplementation and incident autoimmune disease: VITAL randomized controlled trial. BMJ (Clinical Res ed.) (2022) 376:e066452. doi: 10.1136/bmj-2021-066452 PMC8791065 35082139

[32] Grover S Dougan M Tyan K Giobbie-Hurder A Blum SM Ishizuka J . Vitamin d intake is associated with decreased risk of immune checkpoint inhibitor-induced colitis. Cancer (2020) 126:3758–67. doi: 10.1002/cncr.32966 PMC7381363 32567084

[33] Nalluru SS Piranavan P Ning Y Ackula H Siddiqui AD Trivedi N . Hypocalcemia with immune checkpoint inhibitors: The disparity among various reports. Int J Endocrinol (2020) 2020:7459268. doi: 10.1155/2020/7459268 32587615 PMC7294349

[34] Wu X Cheng J Yang K . Vitamin d-related gene polymorphisms, plasma 25-Hydroxy-Vitamin d, cigarette smoke and non-small cell lung cancer (NSCLC) risk. Int J Mol Sci (2016) 17:1597. doi: 10.3390/ijms17101597 PMC5085630 27669215

[35] Hibler EA Klimentidis YC Jurutka PW Kohler LN Lance P Roe DJ . CYP24A1 and CYP27B1 polymorphisms, concentrations of vitamin d metabolites, and odds of colorectal adenoma recurrence. Nutr Cancer (2015) 67:1131–41. doi: 10.1080/01635581.2015.1068818 PMC4589517 26241700

[36] Lopez-Mayorga A Hauger H Petersen RA Vogel U Damsgaard CT Lauritzen L . Vitamin d-related genes and cardiometabolic markers in healthy children: a mendelian randomisation study. Br J Nutr (2020) 123:1138–47. doi: 10.1017/S0007114520000148 31959263

[37] Yu S Feng Y Qu C Huo W Mao Z Wang C . CYP27B1 as an instrument gene to investigate the causal relationship between vitamin d deficiency and obesity: a family-based study. Eur J Clin Nutr (2020) 74:806–10. doi: 10.1038/s41430-020-0594-7 32127688

[38] Liu X Wang G Hong X Wang D Tsai HJ Zhang S . Gene-vitamin d interactions on food sensitization: a prospective birth cohort study. Allergy (2011) 66:1442–8. doi: 10.1111/j.1398-9995.2011.02681.x PMC3189275 21819409

[39] Jiang X O'Reilly PF Aschard H Hsu YH Richards JB Dupuis J . Genome-wide association study in 79,366 European-ancestry individuals informs the genetic architecture of 25-hydroxyvitamin d levels. Nat Commun (2018) 9:260. doi: 10.1038/s41467-017-02662-2 29343764 PMC5772647

[40] Dong J Gharahkhani P Chow WH Gammon MD Liu G Caldas C . No association between vitamin d status and risk of barrett's esophagus or esophageal adenocarcinoma: A mendelian randomization study. Clin Gastroenterol Hepatol Off Clin Pract J Am Gastroenterological Assoc (2019) 17:2227–35.e1. doi: 10.1016/j.cgh.2019.01.041 PMC6675666 30716477

[41] Liu H Jiang X Qiao Q Chen L Matsuda K Jiang G . Association of circulating 25-hydroxyvitamin d and its related genetic variations with hepatocellular carcinoma incidence and survival. Ann Trans Med (2020) 8:1080. doi: 10.21037/atm-20-1637 PMC7575935 33145299

