# Supplementary material for: Vitamin D metabolism pathway polymorphisms are associated with efficacy and safety in patients under anti-PD-1 inhibitor therapy
Source: Front Immunol. 2022 Sep 12;13:937476. doi: 10.3389/fimmu.2022.937476 (PMC9510606; doi:10.3389/fimmu.2022.937476)
Supplement: Supplementary file 1 [file Table_1.docx]

Table 1S. Primers of candidate SNPs.

| SNP ID | 2^nd^-PCRP | 1^st^-PCRP |
| --- | --- | --- |
| rs1544410 | ACGTTGGATGAGAGCAGAGCCTGAGTATTG | ACGTTGGATGGAGGAACTAGATAAGCAGGG |
| rs731236 | ACGTTGGATGCTTCTTCTCTATCCCCGTGC | ACGTTGGATGTGTACGTCTGCAGTGTGTTG |
| rs7975232 | ACGTTGGATGTGCCGTTGAGTGTCTGTGTG | ACGTTGGATGTAGAGAAGAAGGCACAGGAG |
| rs2228570 | ACGTTGGATGTGGCCTGCTTGCTGTTCTTA | ACGTTGGATGACGTTCCGGTCAAAGTCTCC |
| rs2296241 | ACGTTGGATGTCTTCAACGTGGCCTCTTTC | ACGTTGGATGAAATGTGTCTTTTGCGGTTG |
| rs6068816 | ACGTTGGATGCGACTGGAGTGACCATCATC | ACGTTGGATGCTTCCAGAACGAACATTGTC |
| rs2762934 | ACGTTGGATGGTTCCAGAAGCTGTACTGTC | ACGTTGGATGTGTAGAATGCCTTGGATCCC |
| rs10877012 | ACGTTGGATGAGCAGAGAGGTAAACTGTGG | ACGTTGGATGTAGGCAACAGAGAGAGGGC |
| rs2060793 | ACGTTGGATGTGCAGATGGAATTAAAGGGC | ACGTTGGATGGATTATATTGGGCCCACCTG |
| rs7041 | ACGTTGGATGGGCAGAGCGACTAAAAGCAA | ACGTTGGATGGCTTGTTAACCAGCTTTGCC |
| rs12785878 | ACGTTGGATGTCTGGGCTGTCTGATATCAC | ACGTTGGATGAGACGATCAGGCCTTGAGTC |
| rs9409929 | ACGTTGGATGAGCCAACTGCAGACTGTCCA | ACGTTGGATGTTTTGACGTGTCCCCACCCA |
| rs8018720 | ACGTTGGATGTTACCTGTTGGAAGGAGGTG | ACGTTGGATGTCTTTACAGGAAATGCTGGG |

Table 2S. Univariate analysis of clinical features and the curative efficacy.

| Characteristics | Effective, n (%)  (N=241) | Ineffective, n (%) (N=54) | P |
| --- | --- | --- | --- |
| Age, y  mean±SD | 58.70±9.98 | 58.26±11.39 | 0.77 |
| Sex  Male  Femal | 204 (84.65%)  37 (15.35%) | 40 (74.07%)  14 (25.93%) | 0.074 |
| BMI, mean±SD | 22.40±3.63 | 21.36±4.27 | 0.066 |
| Smoke habit | 129 (53.53%) | 22 (40.74%) | 0.099 |
| Drink habit | 73 (30.29%) | 15 (27.78%) | 0.87 |
| Disease stage |  |  |  |
| I-II | 38 (15.77%) | 7 (12.96%) | 0.68 |
| III-IV | 203 (84.23%) | 47 (87.04%) |  |
| Cancer type |  |  |  |
| Non-small-cell lung cancer | 161 (75.23%) | 26 (48.15%) | 0.012 |
| Esophagus cancer | 21 (9.81%) | 3 (5.56%) |  |
| nasopharyngeal carcinoma | 9 (4.21%) | 2 (3.70%) |  |
| Malignant melanoma | 2 (0.93%) | 2 (3.70%) |  |
| Other types | 48 (22.43%) | 21 (38.89%) |  |
| ECOG PS score before treatment |  |  |  |
| 0 | 3 (1.24%) | 2 (3.7%) |  |
| 1 | 230 (95.44%) | 45 (83.33%) | 0.016 |
| ≥2 | 8 (3.32%) | 7 (12.96%) |  |
| Patients with PD-L1 expression level |  |  |  |
| <1% | 51 (29.48%) | 24 (53.33%) | 0.006 |
| 1%-49% | 64 (36.99%) | 8 (17.78%) |  |
| ≥50% | 58 (33.35%) | 13 (28.89%) |  |
| Anti-PD-1 monotherapy | 22 (9.13%) | 12 (22.22%) | 0.016 |
| Treatment line |  |  |  |
| First line therapy | 184 (76.35%) | 29 (53.70%) | 0.001 |
| Second or third line therapy | 57 (23.65%) | 25 (46.30%) |  |
| irAEs | 160 (66.39%) | 27 (50%) | 0.029 |

Table 3s. Association between genotype distribution frequencies of polymorphisms and treatment efficacy of ICBs.

| Gene | SNP | Genotype | Ineffective, n(%)  N=54 | Effective, n(%) N=242 | P |
| --- | --- | --- | --- | --- | --- |
| VDR | rs1544410 | CC  CT  TT | 52(96.30%)  2 (3.70%)  0 (0%) | 219 (90.50%)  21 (8.68%)  1 (0.41%) | 0.40 |
|  | rs731236 | AA  AG  GG  Missing | 51 (94.44%)  3 (5.56%)  0 (0%) | 214 (88.43%)  24 (9.92%)  0 (0%)  4 (1.65%) | 0.43 |
|  | rs7975232 | CC  CA  AA  Missing | 26 (48.15%)  21 (39.89%)  5 (9.26%)  2 (3.70%) | 114 (47.11%)  96 (39.67%)  26 (10.74%)  6 (2.48%) | 1.00 |
| CYP24A1 | rs2296241 | GG  GA  AA  Missing | 21 (38.89%)  28 (51.85%)  4 (7.41%)  1 (1.85%) | 67 (27.69%)  120 (49.59%)  52 (21.49%)  3 (1.24%) | 0.029 |
|  | rs6068816 | TT  CT  CC  Missing | 10 (18.52%)  29 (53.70%)  13 (24.07%)  2 (3.70%) | 97 (40.08%)  118 (48.76%)  20 (8.26%)  7 (2.89%) | 5.10E-04 |
|  | rs2762934 | GG  GA  AA  Missing | 46 (85.19%)  6 (11.11%)  0 (0%)  2 (3.70%) | 185 (76.45%)  51 (21.07%)  2 (0.83%)  4 (1.65%) | 0.21 |
| CYP27B1 | rs10877012 | TT  GT  GG  Missing | 20 (37.04%)  26 (48.15%)  6 (11.11%)  2 (3.70%) | 31 (12.81%)  107 (44.21%)  96 (39.67%)  8 (3.31%) | 0.89 |
| CYP2R1 | rs2060793 | GG  GA  AA  Missing | 23 (42.59%)  24 (44.44%)  7 (12.96%)  0 (0%) | 88 (36.36%)  125 (51.65%)  24 (9.92%)  5 (2.07%) | 0.50 |
| GC | rs7041 | AA  AC  CC  Missing | 25 (46.30%)  24 (44.44%)  5 (9.26%)  0 (0%) | 124 (51.24%)  93 (38.43%)  23 (9.50%)  2 (0.83%) | 0.75 |
| DHCR7 | rs12785878 | GG  GT  TT  Missing | 11 (20.37%)  30(55.56%)  12 (22.22%)  1 (1.85%) | 81 (32.56%)  116(48.84%)  41 (16.74%)  4 (1.65%) | 0.15 |
| RXRA | rs9409929 | GG  GA  AA  Missing | 30 (55.56%)  18(33.33%)  2 (3.70%)  4 (7.41%) | 151 (62.40%)  65(26.86%)  13 (5.37%)  13 (5.37%) | 0.57 |
| SEC23A | rs8018720 | CC  CG  GG  Missing | 18 (33.33%)  28 (51.85%)  7 (12.96%)  1 (1.85%) | 92 (38.02%)  110 (45.45%)  38 (15.70%)  2 (0.83%) | 0.72 |

Table 4s. Patient demographic and baseline characteristics between patients with or without irAEs.

| Characteristics | N-irAEs, n (%) (N=128) | irAEs , n (%) (N=215) | P-Value |
| --- | --- | --- | --- |
| Age, y  mean±SD | 58.36±11.39 | 58.43±10.18 | 0.95 |
| Sex  Male  Femal | 105 (82.03%)  23 (17.97%) | 158 (73.49%)  57 (26.51%) | 0.086 |
| BMI, mean±SD | 22.20±3.21 | 22.41±3.35 | 0.57 |
| Smoke habit | 70 (54.69%) | 108 (50.23%) | 0.44 |
| Drink habit | 37 (28.91%) | 66 (30.70%) | 0.81 |
| Disease stage |  |  |  |
| I-II | 24 (18.75%) | 7 (3.25%) | 6.93E-6 |
| III-IV | 104 (81.25%) | 198 (96.75%) |  |
| Cancer type |  |  |  |
| Non-small-cell lung cancer | 80 (62.50%) | 133 (61.86%) |  |
| Esophagus cancer | 11 (8.59%) | 20 (9.30%) |  |
| nasopharyngeal carcinoma | 7 (5.47%) | 7 (3.26%) |  |
| Malignant melanoma | 1 (0.78%) | 4 (1.86%) |  |
| Other types | 29 (22.66%) | 51 (23.72%) |  |
| ECOG PS score before treatment |  |  |  |
| 0 | 2 (1.56%) | 4 (1.86%) |  |
| 1 | 120 (93.75%) | 200 (93.02%) | 0.96 |
| ≥2 | 6 (4.69%) | 11 (5.12%) |  |
| Patients with PD-L1 expression level | 97 (75.78%) | 148 (68.84%) |  |
| <1% | 35 (36.08%) | 49 (33.11%) | 0.88 |
| 1%-49% | 30 (30.93%) | 49 (33.11%) |  |
| ≥50% | 32 (32.99%) | 50 (33.78%) |  |
| Anti-PD-1plus chemotherapy or radiotherapy | 116 (90.62% ) | 185 (86.05%) | 0.24 |
| Anti-PD-1 monotherapy | 12 (9.38%) | 30 (13.95%) |  |
| Treatment line |  |  |  |
| First line therapy | 91 (71.09%) | 151 (70.23%) | 0.90 |
| Second or third line therapy | 37 (28.91%) | 64 (29.77%) |  |
| Patients with irAEs | 215 (100%) | 0 |  |
| Severe irAEs (grade 3-5) | 56 (26.05%) | 0 |  |
| Mild irAEs (grade 1-2) | 159 (73.95%) | 0 |  |
| No immune-related adverse events | 0 | 128 (100%) |  |

Table 5s. Genotype distribution frequencies of mutations between patients with or without irAEs in this study.

| Gene | SNP | Genotype | N-irAEs, n(%)  N=128 | irAEs, n(%)  N=215 | P |
| --- | --- | --- | --- | --- | --- |
| VDR | rs1544410 | CC  CT  TT  Missing | 119 (92.97%)  7 (5.47%)  1 (0.78%)  1 (0.78%) | 196 (91.16%)  19 (8.84%)  0 (0%)  0 (0%) | 0.69 |
|  | rs731236 | AA  AG  GG  Missing | 115 (89.84%)  12 (9.37%)  0 (0%)  1 (0.78%) | 188 (87.44%)  22 (10.23)  0 (0%)  5 (2.32%) | 0.86 |
|  | rs7975232 | CC  CA  AA  Missing | 59 (46.09%)  56 (43.75%)  11 (8.59%)  2 (1.56%) | 100 (46.51%)  87 (40.46%)  21 (9.77%)  7 (3.25%) | 1.00 |
| CYP24A1 | rs2296241 | GG  GA  AA  Missing | 38 (29.69%)  60 (46.87%)  28 (21.88%)  2 (1.56%) | 63 (29.30%)  110 (51.16%)  40 (18.60%)  2 (0.93%) | 0.75 |
|  | rs6068816 | CC  CT  TT  Missing | 40 (31.25%)  69 (53.91%)  15 (11.72%)  4 (3.13%) | 84 (39.07%)  98 (45.58%)  26 (12.09%)  7 (3.26%) | 0.32 |
|  | rs2762934 | GG  GA  AA  Missing | 109 (85.16%)  19 (14.84%)  0 (0%)  0 (0%) | 160 (74.42%)  47 (21.86%)  2 (0.93%)  6 (2.79%) | 0.051 |
| CYP27B1 | rs10877012 | TT  GT  GG  Missing | 61 (47.66%)  49 (38.28%)  11 (8.59%)  7 (5.47%) | 72 (33.49%)  110 (51.16%)  30 (13.95%)  3 (1.40%) | **0.0057** |
| CYP2R1 | rs2060793 | GG  GA  AA  Missing | 52 (40.63%)  63 (49.22%)  11 (8.59%)  2 (1.56%) | 76 (35.35%)  111 (51.63%)  25 (11.63%)  3 (1.40%) | 0.83 |
| GC | rs7041 | AA  AC  CC  Missing | 68 (53.13%)  49 (38.28%)  11 (8.59%)  0 (0%) | 105 (48.84%)  84 (39.07%)  23 (10.70%)  3 (1.40%) | 0.44 |
| DHCR7 | rs12785878 | GG  GT  TT  Missing | 38 (29.69%)  68 (53.13%)  21 (16.41%)  1 (0.78%) | 70 (32.56%)  105 (48.84%)  36 (16.74%)  4 (1.40%) | 0.75 |
| RXRA | rs9409929 | GG  GA  AA  Missing | 81 (63.28%)  37 (28.91%)  3 (2.34%)  7 (5.47%) | 130 (60.47%)  62 (28.84%)  13 (6.05%)  10 (4.65%) | 0.27 |
| SEC23A | rs8018720 | CC  CG  GG  Missing | 54 (42.19%)  50 (39.06%)  23 (17.97%)  1 (0.78%) | 74 (34.42%)  113 (52.56%)  26 (12.09%)  2 (0.93%) | 0.041 |
